# The invasive potential of *Giardia intestinalis* in an *in vivo* model

**DOI:** 10.1038/srep15168

**Published:** 2015-10-16

**Authors:** R. Reynoso-Robles, M. Ponce-Macotela, L. E. Rosas-López, A. Ramos-Morales, M. N. Martínez–Gordillo, A. González-Maciel

**Affiliations:** 1 Laboratorio de Morfologia Celular y Tisular (Laboratory of Cell and Tissue Morphology), Instituto Nacional de Pediatría (Mexican National Institute of Paediatrics), Insurgentes Sur No. 3700-C, Mexico, D. F. C. P. 04530, Mexico; 2 Laboratorio de Parasitología Experimental (Laboratory of Experimental Parasitology), Instituto Nacional de Pediatría (Mexican National Institute of Paediatrics).

## Abstract

Giardiasis is a neglected parasitic disease that affects primarily children, in whom it delays physical and mental development. The pathophysiology of giardiasis in not well understood, and most reports have identified *Giardia intestinalis* trophozoites only in the lumen and on the brush border of the small intestine. We identified *Giardia* trophozoites within the epithelium of the small intestine of a lactose intolerance patient. The *Giardia* trophozoites were obtained and cultured *in vitro*. In addition, we demonstrated *Giardia* trophozoite invasion in an animal model. *Giardia* trophozoites invaded the intestinal mucosa and submucosa of infected gerbils. The invasive trophozoites were observed at 21, 30 and 60 days age, and the average numbers of invaded sites were 17 ± 5, 15 ± 4, and 9 ± 3, respectively. We found trophozoites between epithelial cells, at the base of empty goblet cells, in lacteal vessels and within the submucosa. The morphological integrity of the invasive trophozoites was demonstrated via electron microscopy. The analysis of the gerbils infected with the trophozoites of the WB reference strain did not show intraepithelial trophozoites. These results demonstrate another *Giardia* pathogenic mechanism, opening the door to numerous future studies.

*Giardia intestinalis* (Syn. *G. duodenalis or G. lamblia*), is a protozoan flagellate that parasitizes humans and animals and is transmitted by the ingestion of food or water contaminated with cysts or via person-to-person contact^1^. Giardiasis was included in the “Neglected Diseases Initiative” in 2004^2^. In the underdeveloped world alone, nearly 300 million infections are estimated to occur per year^3^. In Mexico an epidemiologic analysis showed a seroprevalence of 55% in the general population^4^. Although *G. intestinalis* is more prevalent in places with poor sanitation, it is common throughout the world due to the globalisation of travel and the food supply, lifestyle factors, and climate change^5^,^6^. Clinical manifestations of *G. intestinalis* infections vary among individuals, ranging from acute to chronic infection, whereas some hosts are asymptomatic. Patients with acute giardiasis present with abdominal pain, foul smelling explosive watery diarrhoea, steatorrhoea, vomiting and nausea. Patient with chronic giardiosis present with abdominal pain, diarrhoea, weight loss and malabsorption^7^,^8^,^9^. When giardiasis occurs during the first months of life, it affects the growth and cognitive function of newborns^10^,^11^,^12^. Studies of humans and animal models, have shown that *Giardia* trophozoites do not invade the intestinal tissue; they inhabit only the lumen and the microvilli of the intestine, where they closely associate with the intestinal epithelium and initiate pathophysiological changes^13^,^14^,^15^,^16^,^17^,^18^,^9^. Few reports have shown trophozoites inside the mucosa and submucosa^19^,^20^,^21^,^22^. We found morphologically intact *Giardia* trophozoites within the duodenal epithelium of a biopsy from a patient with lactose intolerance and chronic abdominal pain. The *Giardia* trophozoites were obtained and cultured. The *Giardia* isolate was designated INP220806-HGINV (Human *Giardia* Invader HGINV) and genotyped; it belonged to assemblage A, genetic group A2^23^. We hypothesized that the ability of these trophozoites to invade human duodenal tissue, would be recapitulated in an experimental animal model. The present study aimed to verify that trophozoites of *G. intestinalis* HGINV could enter the duodenal tissue of gerbils.

## Results

### Light microscopy

The ability of *Giardia* to invade tissue was successfully demonstrated in a gerbil model. We identified trophozoites in the mucosa and submucosa of 75% of gerbils inoculated with the HGINV *Giardia* isolate at 21, 30 and 60 days of age. Invasion was observed starting on day 18 post inoculation (p.i.) and up to 57 days p. i.; the times correspond to the ages at the time of duodenum analysis gerbils 21, 30 and 60 days of age.

**Figure 1:**
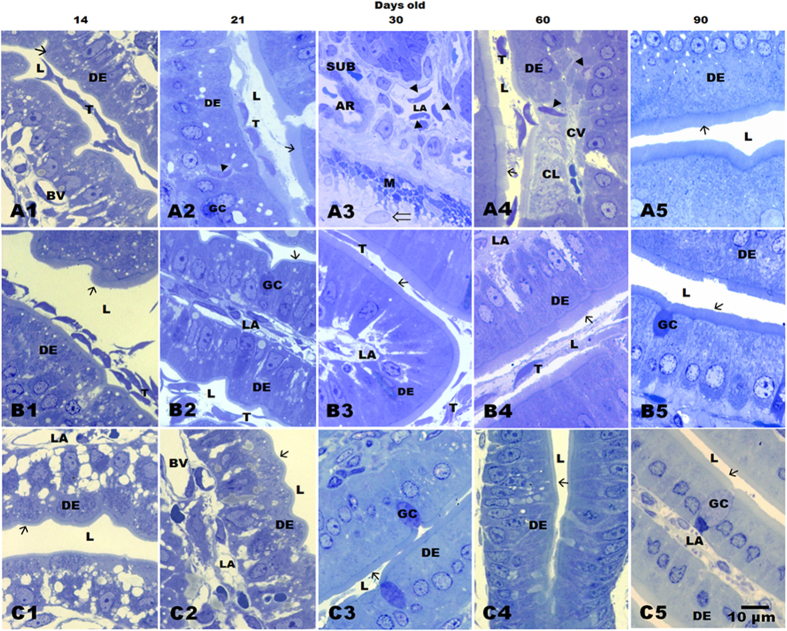
Light microscopy images of the duodenal villi of the gerbils at different ages: gerbils inoculated with the HGINV isolate (**A1–A5**), the WB reference isolate (B1–B5) and the control group (**C1–C5**). A2 shows a trophozoite with a typical crescent shape embedded in the tissue at the level of the enterocytes nuclei. A3 shows three trophozoites inside a lacteal vessel. A4 shows damage to the epithelium and the nuclei of cells in that area; additionally one trophozoite is entering at the centre of the villus (CV) and another is already inside. The epithelium had regenerated by 90 days (A5); inoculation with WB did not damage the epithelium (B1–B5). *Giardia* trophozoites embedded in the tissue (arrowheads); trophozoites (T); Lumen (L); brush border (→); duodenal epithelium (DE); goblet cells (GC); lacteal vessel (LA); submucosa (SUB); muscular (M); myenteric plexus neuron (⇒); blood vessel (BV); arteriole (AR); cell lysis (CL). Sections (0.5 μm) stained with toluidine blue; 100X; scale bar 10 μm. Acknowledged to Gonzalez-Maciel A. by images.

The number of trophozoites on the brush border or in the lumen was higher in 14 and 30 days old gerbils infected with the HGINV isolate than in gerbils of the same age inoculated with a WB reference strain, p < 0.01 (Table 1). We also found 17 ± 5, 15 ± 4 and 9 ± 3 invasion sites in 21, 30 and 60 day gerbils infected with invasive *Giardia*, respectively. Interestingly, invasion sites persisted in 60 day gerbils infected with HGINV, when the population of *Giardia* was nearly eliminated (Table 1). Parasites were embedded in the villous epithelium, inside lacteal vessels, in the submucosa, and near the muscle fibres (Figs 1A2). Damage on the brush border was observed only in some places as shown in the tissue sections of 21 and 60 days old gerbils. In addition we observed nuclei loss in epithelial cells, although evidence of inflammatory reaction was absent from all samples (Fig. 1A2–A4). In general, the integrity of the intestinal tissue was preserved (Figs 1A1).

The infection was eliminated by 90 days of age and both groups, and the epithelium exhibited regeneration (Fig. 1A5 and B5; Table 1). In samples from animals inoculated with the WB reference strain, trophozoites were found only in the lumen, without damage to the epithelium (Fig. 1B1–B4), and control gerbils showed normal duodenal tissue that was free of parasites (Fig. 1C1–C5).

### Electron microscopy

**Figure 2:**
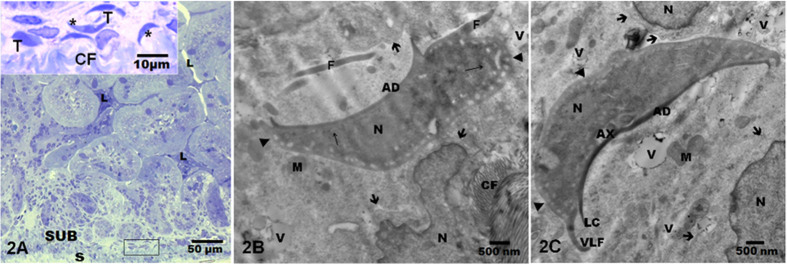
(**A**) Low magnification of the duodenal epithelium of 21 day-old gerbils inoculated with HGINV isolate. Trophozoites in the submucosa (box) are shown at higher magnification on top. 2B and 2C show electron microscopy images of the top trophozoites denoted whit an asterisk (*). The morphology appeared normal. One nucleus (N) of connective tissue cells in proximity to the trophozoites showed nuclear or cytoplasmic membrane integrity (thick arrows) and preserved mitochondria (M). Serosa (S); lumen (L); vacuoles (V); collagen fibres (CF); adhesive disc (AD); lateral crest (LC); ventrolateral flange (VLF); flagellum (F), axonemes (AX). 2A: 20x toluidine blue; on top 100X. 2B and 2C: 15,000 x. Figure 2A: Acknowledged to Gonzalez-Maciel A. by images. Figure 2B,C: Acknowledged to Reynoso-Robles R. by images.

**Figure 3:**
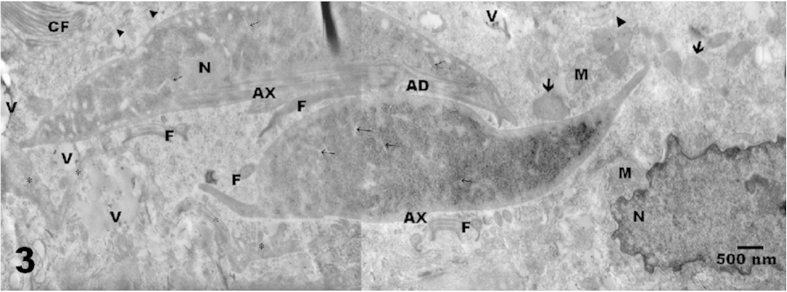
Trophozoites from the HGINV isolate with a normal ultrastructure found in a 30-day-old gerbil. Abundant glycogen granules and ribosomes showed a characteristic electron-dense appearance. The parasites were integrated into the connective tissue of the submucosa. Extensions of the fibroblast membranes (*) and an intact basal membrane (arrowheads) were observed surrounding the trophozoites. Vacuoles (V); endoplasmic reticulum (thin arrows); mitochondria (M), electron-dense granules (arrows); collagen fibres (CF); adhesive disk (AD); flagella cut in various planes (F), axonemes (AX); cell nucleus (N). 20000x. Figure 3 Acknowledged to Reynoso-Robles R. by images.

The ultrastructural analysis revealed intact trophozoites within the submucosa (Fig. 2A). These trophozoites contained vacuoles in their dorsal and cytoplasmic membranes and endoplasmic reticulum, which may be result of their metabolic activity as previously reported^24^,^25^,^26^. In addition trophozoites characterized by a typical morphology were found between the nuclei of connective tissue cells, mitochondria, vacuoles, collagen fibres and electron-dense granules (Figs 2B,C and 3).

### Immunocytochemistry

**Figure 4:**
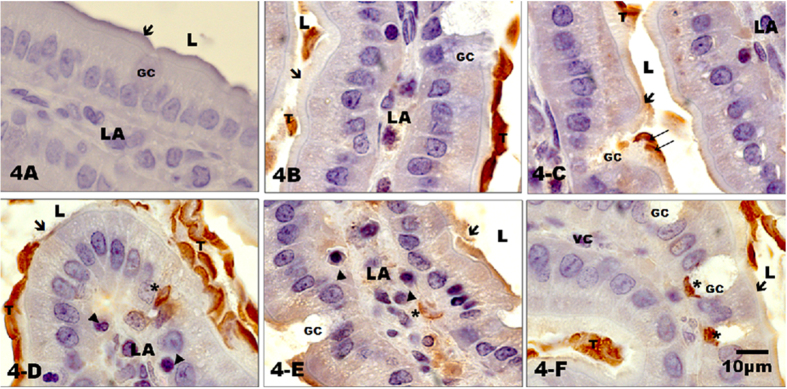
Immunocytochemistry against *Giardia intestinalis* in the duodenal villi of gerbils at 30 days of age. (4A) Control animal; (4B) infection with the reference isolate WB; (4C to 4F) infection with the HGINV isolate. The duodenal epithelium from the controls and those infected with the WB isolate exhibited a normal appearance with an intact brush border (arrows). WB (4B) trophozoites were found on the brush border (T). HGINV trophozoites were found heading toward the interior epithelium (4C), between the nuclei of epithelial cells (4D), in the centre of the villi in the lacteal vessels (4E), and at the base of empty goblet cells (4F). Lumen (L); trophozoites inside the intestinal epithelium (*); lacteal vessels (LA); goblet cell (GC); lymphocyte (arrow head). Counter-staining with haematoxylin, 100x. Figure 4 Acknowledged to Gonzalez-Maciel A. by images.

The immunocytochemistry of *Giardia* revealed trophozoites that were dyed a deep brown colour. No trophozoites were found in the gerbils of the control group (Fig. 4A), whereas trophozoites were found only on the brush border in gerbils infected with the WB reference strain (Fig. 4B). In gerbils inoculated with the HGINV *Giardia* isolate we observed trophozoites in the broken brush border, at the base of empty goblet cells, embedded between the intestinal epithelium cells and in the middle of lacteal vessels (Fig. 4C–F).

Diarrhoea was not detected in any of the cases during the course of the study, but the body weights of the gerbils inoculated with the WB reference strain and the HGINV isolates were lower than that the body weight of the gerbils of the control group at 14, 21 and 30 days age, (p < 0.01). The weight loss was similar between gerbils infected with WB or HGINV (Table 2).

## Discussion

Studies of humans and animal models have shown that *Giardia* trophozoites do not invade intestinal tissue, and the pathophysiology of giardiasis only occasionally includes increased intraepithelial lymphocytes and partial villous atrophy^7^,^9^.

In contrast the results reported herein demonstrate that the invasive potential of the HGINV isolate is similar to that reported in other study^23^. We hypothesize that another trophozoites may be invasive, and previous studies that found trophozoites in duodenal tissue^19^,^20^,^21^,^22^ likely did not identify other invasive trophozoites due to the faulty handling of samples, as has been previously reported^23^. Indeed, the methodology used in this work, specifically the use of a reference isolate as a control that showed no invasion or damage to the villi, rules out the presence of methodological error and highlights the invasive potential.

In accordance with Amorim *et al.* and Belosevic *et al.*, the gerbil is considered the best experimental model to study the development and pathogenesis of *G. lamblia* infections. Specifically, the gerbil is highly susceptible to *G. lamblia* infection via the oral inoculation of cysts and trophozoites, and many cysts are eliminated in the stool, thus eliciting the same pathophysiological alterations observed in humans^27^,^28^. The gerbil model does not mirror human disease because the clinical manifestations of *G. intestinalis* infection are known to vary among individuals, ranging from acute to chronic infection, whereas some hosts are asymptomatic. The clinical symptoms and pathophysiology of giardiasis result from a combination of both the host and parasitic factors which have yet to be identified^9^,^29^. These interactions may result in absence of diarrhoea and the morphological absence of inflammation. With respect to the former, a consistent softening of the faeces has only been observed with assemblage B^30^, and impaired weight gain is the most common sign of *Giardia* infection^31^. However, studies have attested to a deficiency of CD5^+^ B- lymphocytes and responses deficiency Th1-Th2 in gerbils^32^,^33^. Moreover, *Giardia* trophozoites can attenuate the production of inflammatory mediators from intestinal tissues of differing origins, thus supporting the idea that infection with specific *Giardia* isolates may modulate host immune responses in the gut^34^.

The results of our study indicate a similarity between isolates of *G. intestinalis* in their ability to colonize the duodenum of gerbils. It is noteworthy that the dose of inoculum was same for the WB and the invasive strain, and that the number of trophozoites in the lumen was similar, differing only in two of the five times it was assessed. The presence of invading trophozoites in 60 days old gerbils was surprising, since the population of trophozoites was nearly eliminated in specimens of that age. The fate of the invading trophozoites remains unclear because the observations were made at isolated time points.

The current data suggest that this invasive isolate may utilize more than one mechanism to penetrate the duodenal tissue. Based on these morphological observations, this isolate may take advantage of the epithelial discontinuity resulting from the mucus discharge from the goblet cells, as was described elsewhere^35^. Alternatively, it may actively secrete substances that facilitate invasion. The presence of the protozoa at the base of the goblet cells suggests that this site could be a route of entry. The base of these cells is only a few microns from the centre of the villus. From entry at the base of the cells, *Giardia* could pass through the lamina propria and migrate to deeper sites of the villi. The presence of trophozoites in the lacteal vessels may suggest that the parasites travel from the lacteal vessels to other tissues, as reported by el-Shewy and Eid^36^, who identified *Giardia muris* in the renal tissue of naturally infected mice.

*In vitro* studies showed that *Giardia* trophozoites alter their paracellular permeability, induce apoptosis, and reorganize the cytoskeletal proteins associated with tight junctions, adherent junctions, and desmosomes upon adhering to the surface of epithelial cells^37^. These effects have also been suggested to be due to the movements of the trophozoites on the villus during the adhesion or release of toxins by the parasite^15^,^37^, although additional studies are necessary to support this hypothesis.

The medical literature details few cases of invasive giardiasis and these cases have generally been attributed to the mismanagement of the sample rather than an invasion process^23^. In our previous work the patient was treated with tinidazole, and subsequent tests showed that lactose absorption was normal, stool examinations were negative for *Giardia* and abdominal pain had stopped. These results suggest that the symptoms may be related to intraepithelial giardiasis. Therefore this study reveals another *Giardia* pathogenic mechanism and opens the door to future research; clearly the next steps are to address differences in the genetics and transcriptome to see what may be underlying these differences.

The analysis of this case and the review of the literature suggest that patients with steatorrhea, abdominal pain, or lactose malabsorption may be harbouring invasive trophozoites, although further studies are required to confirm this assessment.

To our knowledge, the present study is the first to demonstrate the phenomenon of tissue invasion by *Giardia* trophozoites in an *in vivo* model. Our study shows that of the two strains used, only the HGINV strain has invasive potential.

## Methods

All animal procedures were approved by the ethics committee of the National Institute of Paediatrics in accordance with the provisions of the National Institutes of Health (Institutos Nacionales de Salud, NOM-062-ZOO-1999).

### Giardia culture

Trophozoites of isolate INP220806-HGINV (HGINV), which belong to assemblage A, genetic group A^23^, and the WB strain reference (genetic group A-1) were axenic-cultured in TYI-S-33 medium, harvested in the log phase, washed in phosphate-buffered saline (PBS, 0.1M, pH 7.0), and counted in a Neubauer chamber to obtain aliquots for the inoculation of gerbils^38^.

### Animals

**Figure 5:**
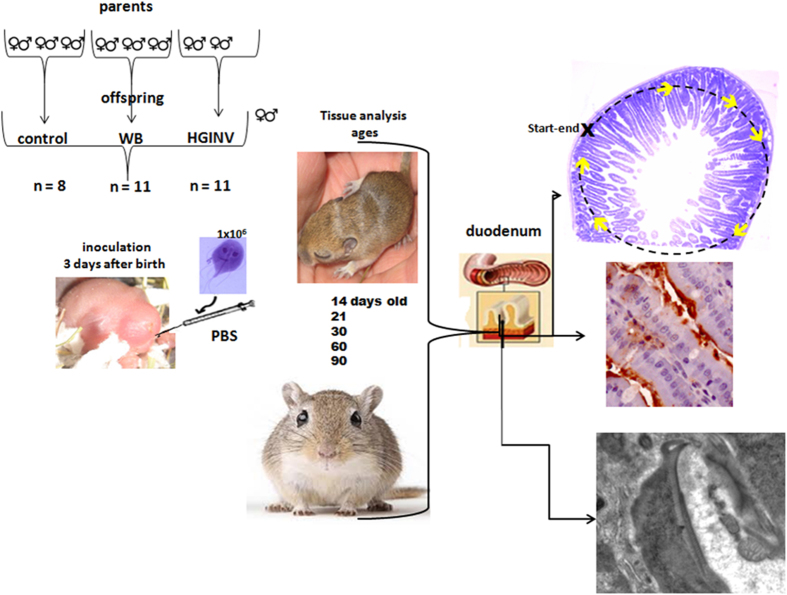
Strategy to assess the invasive potential of *Giardia intestinalis* in an *in vivo* model. Shows study groups, ages and methodology. Figure 5 Acknowledged to Gonzalez-Maciel A. by photographs and drawings. Thanks to Elsevier, for Scheme of the duodenum, license number 3697761086894. Experimental Parasitology, 133(4).

*Meriones unguiculatus* were obtained from the bioterium of the National Institute of Nutrition “Salvador Subiran”. The animals were housed in plexiglass boxes under standard vivarium conditions: a 12:12 light/dark cycle, 40% humidity, and controlled temperature (23 ± 3°C). The gerbils were provided with filtered water and commercial rodent chow *ad libitum.* All food was autoclaved, and their diet was supplemented with previously disinfected sunflower seeds and carrots. To obtain offspring, we formed 9 pairs of unrelated gerbils of 45 days of age that were free of pathogens and divided the pairs into 3 groups of 3 pairs (Fig. 5). Daily vaginal smears were taken from day 70 to determine the onset of gestation, and; the presence of sperm indicated day one.

### Gerbils infected with *Giardia* trophozoites

The experimental groups included three or four animals from each litter. The following equation was used to calculate the number of gerbils per group: n = (Z ∝ δ/E)^2^, where δ = 32.33, Z = 1.96, α = 0.025, and E = 15, with an infection average of 88.88%. The control group contained 8 animals, and the experimental animal groups of each age contained 11 animals per group. The offspring were infected orally 3 days after birth via gavage with 1 × 10^6^ trophozoites from the HGINV or WB isolates in 50 μl of PBS (0.1 M, pH 7.4), which was used as a vehicle. The control offspring received 50 μl of PBS. The duodenum of the gerbils was assessed 11, 18, 27, 57 and 87 days post inoculation p. i. These times corresponded to 14, 21, 30, 60, and 90 days of age (Fig. 5). The animals were weaned on day 21, and the remaining offspring from the different litters of the same condition were placed in boxes until they reached 30, 60, and 90 days of age. The body weight of each gerbil was recorded before euthanasia.

### Preparation of tissue

At the indicated ages, the gerbils were euthanized; with an overdose of chloral hydrate (1 ml/kg body weight). They were then transcardially perfused with a cold solution of PBS (0.1M pH 7.4) followed by a mixture of glutaraldehyde fixative (2%) and paraformaldehyde (4%) in PBS. Subsequently, 2 cm of the duodenum of each animal was collected. One piece of tissue was embedded in epoxy resin (Epon) for light and electron microscopy analyses, and the other piece was embedded in paraffin for the immunocytochemical detection of *Giardia* trophozoites (Fig. 5).

### Evaluation of invasion sites and trophozoites among the villi

Using the material embedded in Epon, ten 0.5-μm transverse sections, with a separation of 20 μm between cuts, were obtained to ensure that the trophozoites observed in each section differed (the average diameter of a *Giardia* trophozoite is 10–12 μm). The sections were obtained using a Leica ultramicrotome EM UC6 (Solms, Germany). The sections were stained with toluidine blue and the thickness and staining allowed trophozoites to be cleatly visualized among the villi and/or within the epithelium. The tissues were observed using an Axioscop 2 Plus microscope coupled to an image analysis system (Axiovision software, Version 4.6), equipped with a 100× objective (Carl Zeiss). The number of trophozoites among the villi in a constant area (14.000 μm^2^) was estimated in 5 fields of each section for each animal. To detect the invasion sites, which were defined as one or more trophozoites identified among the villi in either the mucosa, submucosa or muscle, all villi in each section were reviewed. A reference point for the start of the observation was selected, and the tissue was scanned in the clockwise direction until the starting position was reached.

A well-trained person blinded to the samples performed all assessments by assigning a numerical code to each slide. The results are reported as the mean and standard deviation. An analysis of variance (ANOVA) and a comparison of means using a Tukey’s test (statistical program inerst13) were performed. To assess the status of the trophozoites internalized in the tissue, 60–90-nm sections were obtained and collected on Formvar-covered grids contrasted with uranyl acetate and lead citrate. The observations were made using a JEOL-1011 transmission electron microscope (Osaka, Japan).

### Antibodies

Polyclonal antibodies directed against the trophozoites of the HGINV isolate were generated by inoculating the peritoneum of a rat with one million live trophozoites and boosting this inoculation two times every 15 day. Rat IgG antibodies were purified via column chromatography and packed with protein A-agarose (Roche diagnostics GmbH), according to the instructions of the provider. The purified antibodies were quantified using ELISA^39^, tested and then stored at –20 °C in 50% glycerol until use.

### Immunocytochemistry against *Giardia* trophozoites

Immunocytochemistry was used to detect *G. intestinalis* trophozoites in sections of 5 μm thickness. The sections were deparaffinized and rehydrated. Antigen were recovered with 0.1 M citrate buffer at pH 6.0, and endogenous peroxidase activity was blocked using hydrogen peroxide (1.6% in methanol). The sections were incubated for 2 hours in 10% normal goat serum (Vector Laboratories, Inc., Orton Southgate, United Kingdom) followed by incubation with a primary antibody against *Giardia* trophozoites (1:1,000, developed against trophozoites and their excretory products in rats at the laboratory of Experimental Parasitology of the National Institute of Paediatrics. Samples were diluted in PBS (0.1 M, pH 7.4). Subsequently, the avidin-biotin-peroxidase method (ABC kit, Vector Laboratories, Inc., Orton Southgate, United Kingdom) was used, and the reaction was revealed with the chromogen 3′3′-diaminobenzidine (Sigma Aldrich, St. Louis, USA). The samples were counterstained with haematoxylin (H), and the sections were then mounted in resin (Entellan, Merck, Darmstadt, Germany).

## Additional Information

**How to cite this article**: Reynoso-Robles, R. *et al.* The invasive potential of *Giardia intestinalis* in an *in vivo* model. *Sci. Rep.* **5**, 15168; doi: 10.1038/srep15168 (2015).

## Figures and Tables

**Table 1:** Number of trophozoites among the villi of the duodenum in gerbils inoculated with trophozoites of the WB and HGINV isolates.

Age in days	Wb isolate # of trophozoites	Invasion sites	HGINV isolate # of trophozoites	Invasion sites
14	29 ± 8	0	40 ± 5*	0
21	31 ± 4	0	37 ± 3	17 ± 5
30	19 ± 3	0	45 ± 6*	15 ± 4
60	6 ± 2	0	9 ± 3	9 ± 3
90	0	0	0	0

Only HGINV parasites entered the tissue. The HGINV population was significant (p < 0.01*).

**Table 2:** Body weight of the control gerbils and the gerbils inoculated with trophozoites of the HGINV and WB isolates at different ages.

Age in days	Control Body weight (grams)	WB isolate Body weight (grams)	HGINV isolate Body weight (grams)
14	15 ± 0.7	10 ± 1.0*	11 ± 1.0*
21	20 ± 0.5	15 ± 2.7*	15 ± 1.1*
30	32 ± 3.3	21 ± 1.5*	23 ± 3.4*
60	54 ± 2.0	52 ± 3	51 ± 2.1
90	67 ± 3.5	64 ± 4.1	71 ± 5.4

Mean value and SD. The weights of the inoculated gerbils were significantly lower (*p < 0.01) than the weights of the controls animals of the same age.
